# Supplementary material for: Modularity of genes involved in local adaptation to climate despite physical linkage
Source: Genome Biol. 2018 Oct 5;19:157. doi: 10.1186/s13059-018-1545-7 (PMC6173883; doi:10.1186/s13059-018-1545-7)
Supplement: Supplementary file 1 — Supplementary Figures. Fourteen supplementary figures associated with the manuscript. (PDF 12466 kb) [file 13059_2018_1545_MOESM1_ESM.pdf]

Supplementary Material

KE Lotterhos, S Yeaman, J Degner, S Aitken, and KA Hodgins. Modularity of genes involved in local adaptation to climate despite physical linkage. Genome Biology.

Contents

List of Figures 1

1. Supplementary Figures 2

List of Figures

1 S1 $X^T X$ histogram for outliers 2

2 S2 Enlarged Multi network 3

3 S3 Enlarged Aridity network 4

4 S4 Enlarged Freezing network 5

5 S5 Enlarged Geography network 6

6 S6 Structure-corrected heatmap 7

7 S7 Linkage disequilibrium heatmap 8

8 S8 Recombination heatmap, clustered by recombination rates 9

9 S9 Loadings of environments onto PC axes 10

10 S10 Outliers on PC axes 11

11 S11 SNP annotations and genomic features 12

12 S12 Box plots of error rates in simulations 13

13 S13 Pairwise distances among loci as a function of selection for simulated data 14

14 S14 More examples of networks from simulations 15

1. Supplementary Figures

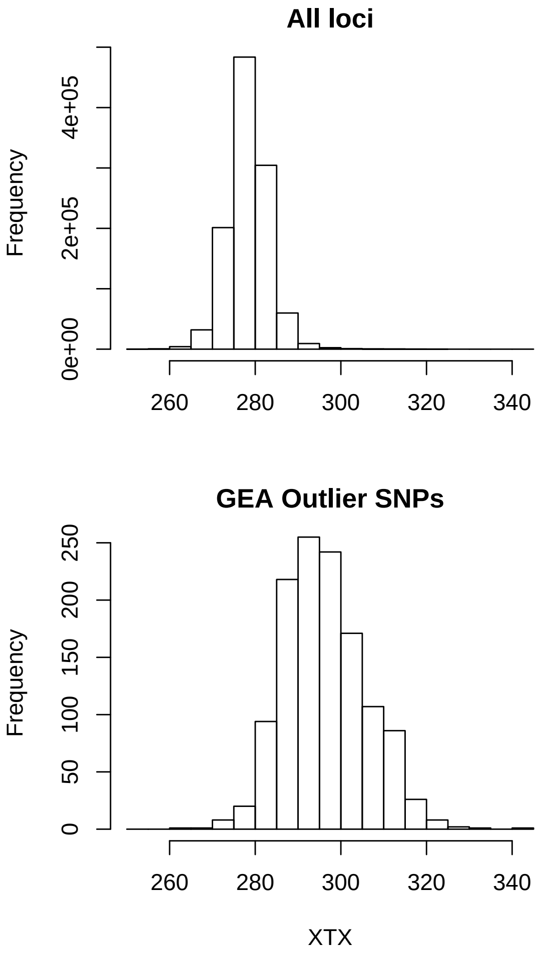

Figure S 1. Histogram of X^TX estimated from Bayenv2 for all SNPs (top) and for top candidate SNPs (bottom) from the gene-environment association (GEA).

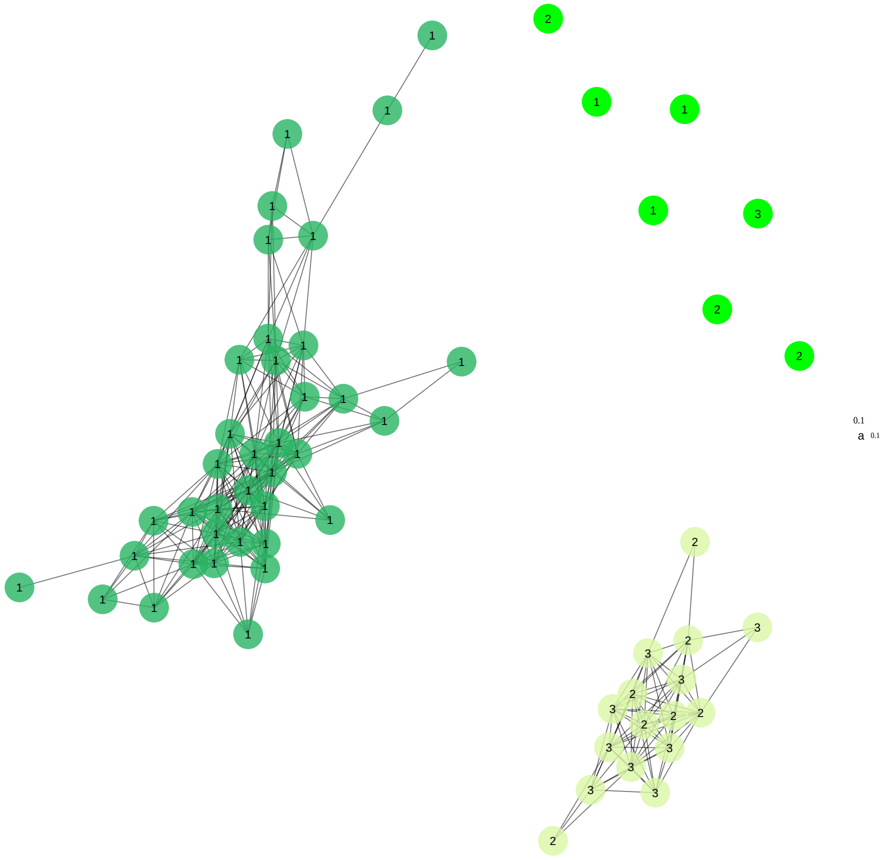

Figure S 2. Undirected graph network for the Multi group (enlarged version of Figure 2C).

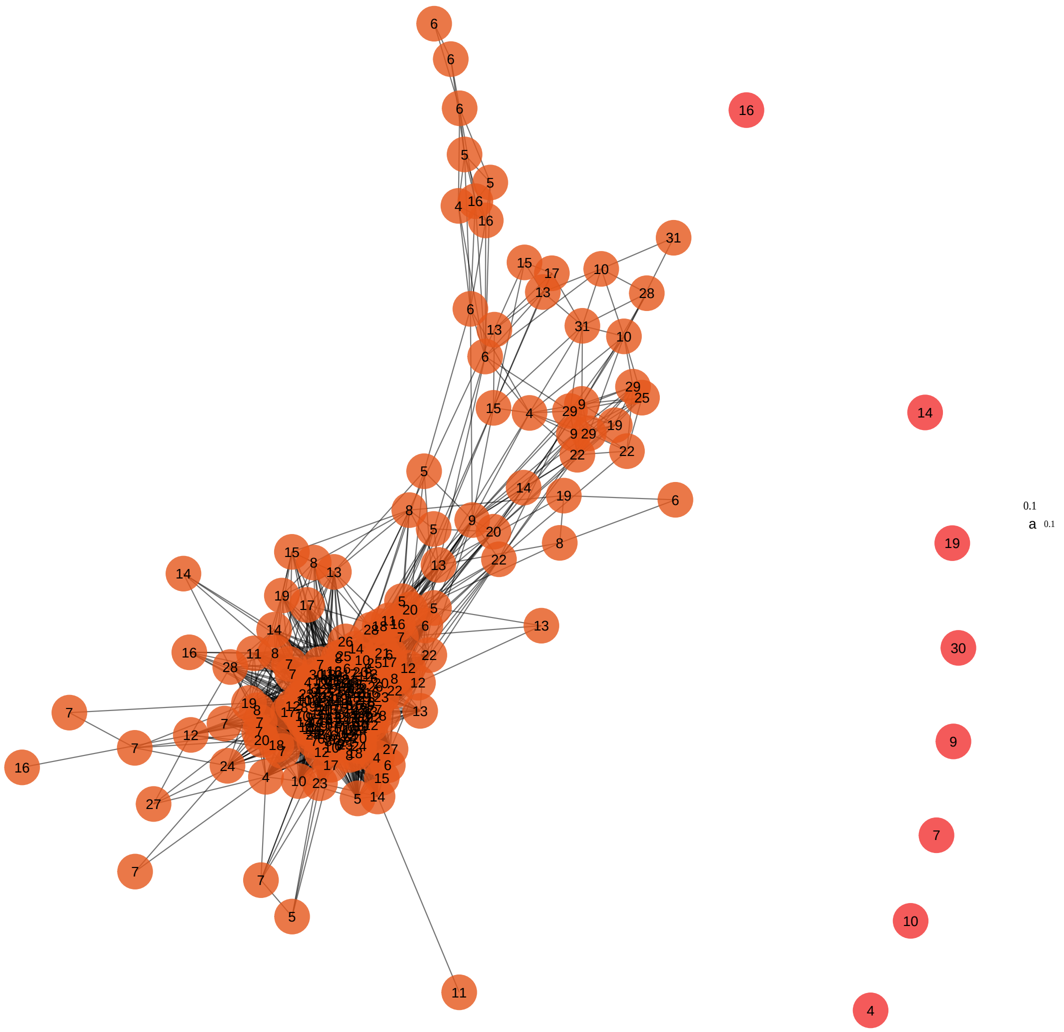

Figure S 3. Undirected graph network for the Aridity group (enlarged version of Figure 2D).

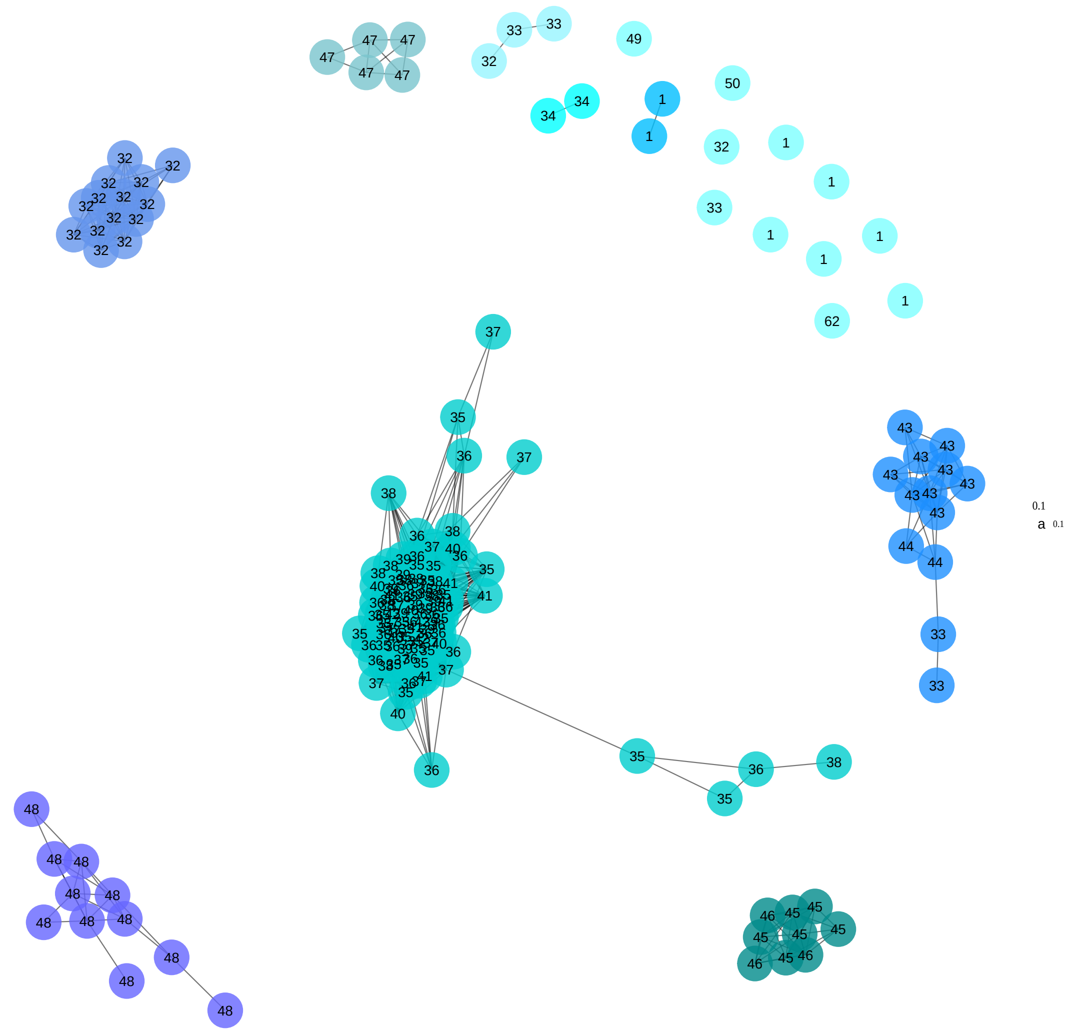

Figure S 4. Undirected graph network for the Freezing group (enlarged version of Figure 2E).

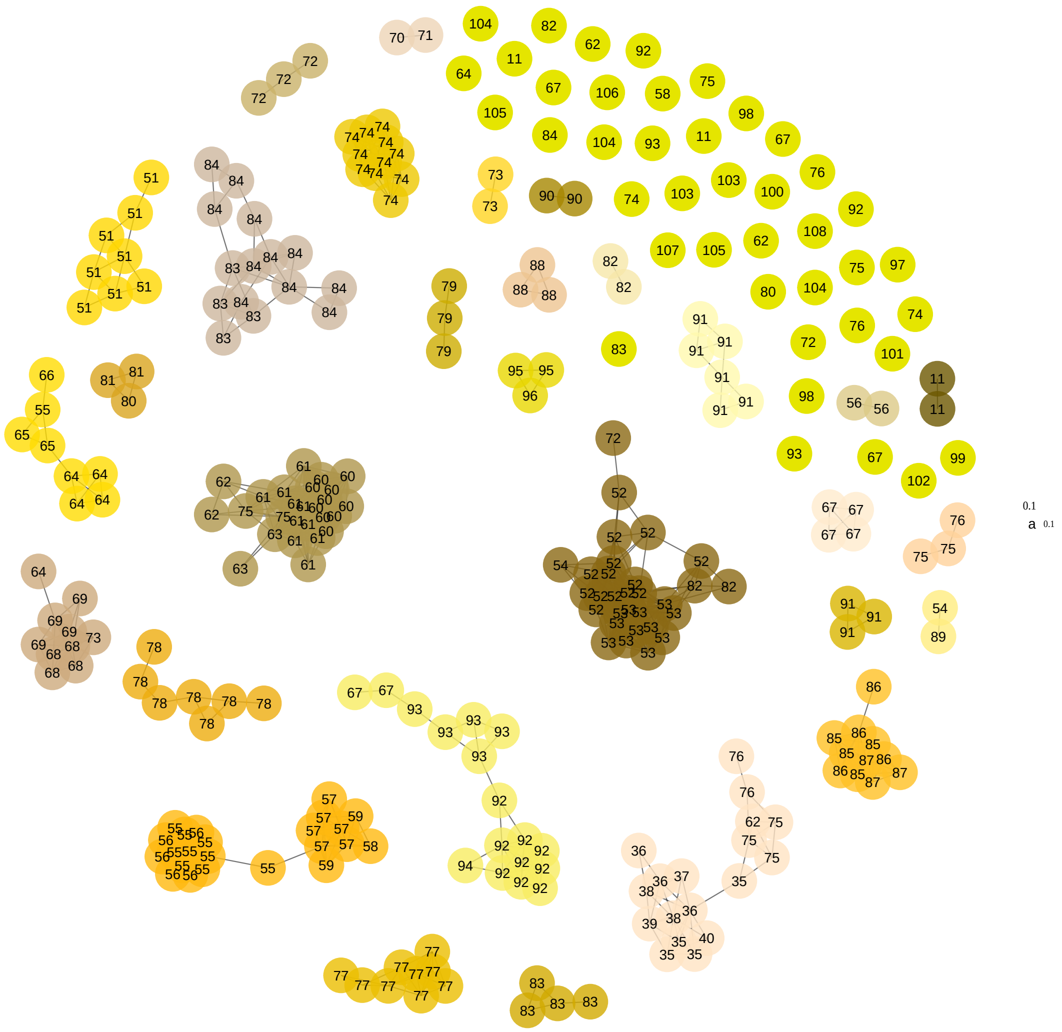

FIGURE S 5. Undirected graph network for the Geography group (enlarged version of Figure 2F).

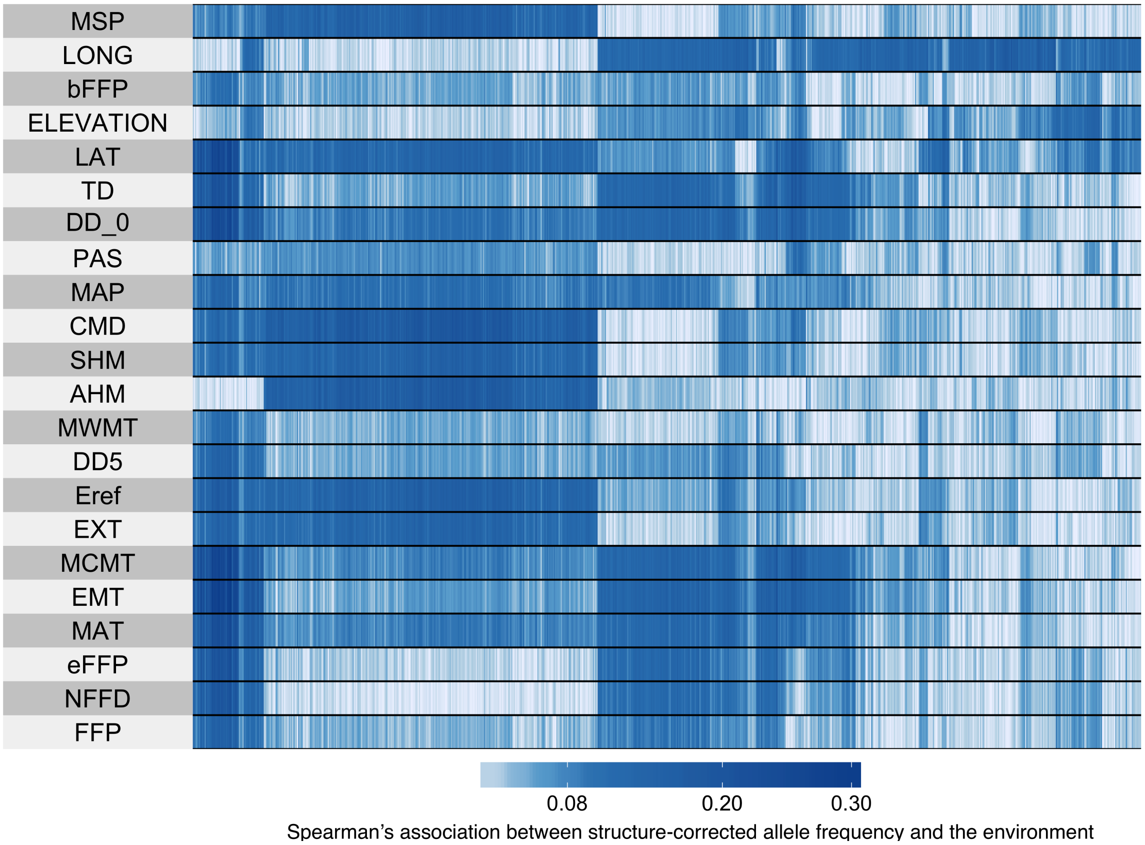

Figure S 6. Heatmap of structure-corrected absolute value of allele associations with the environment, analogous to Figure 2B in the main paper. Note that although the pattern is very similar, the magnitude of allele correlations is smaller in the structure-corrected data (scale of 0 to 0.3 here, compared to 0 to 0.5 for uncorrected associations in Figure 2B).

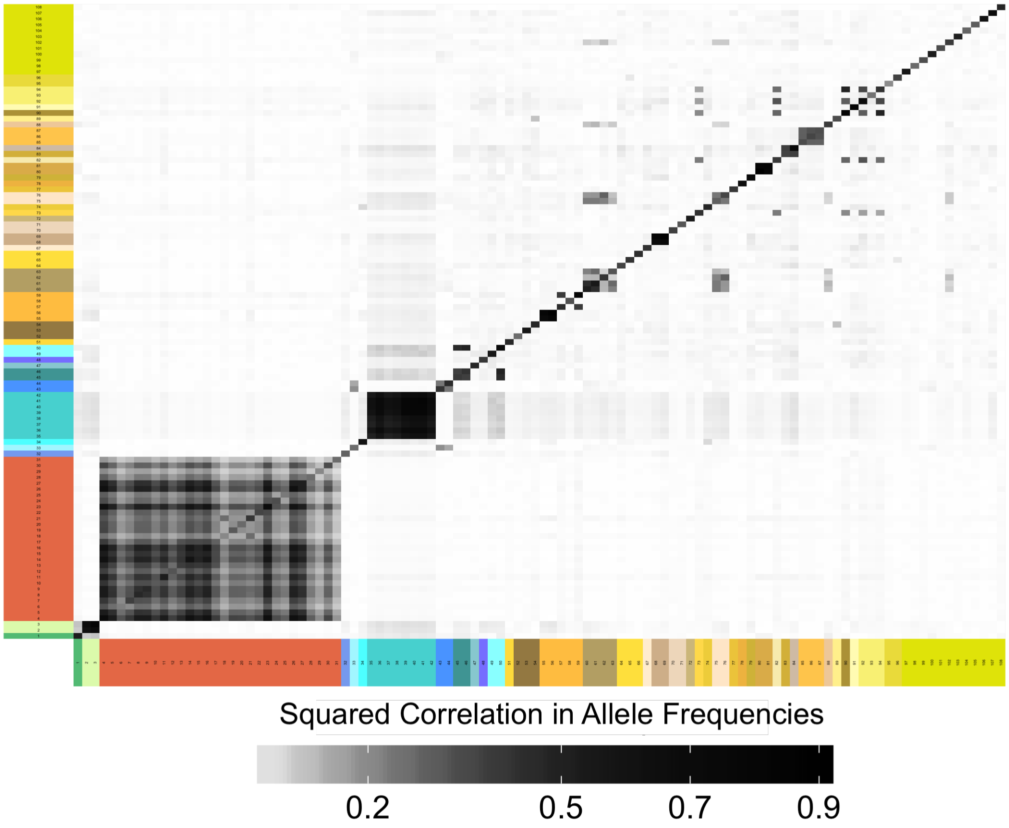

Figure S 7. Linkage disequilibrium heatmap. Mean squared correlation among allele frequencies between all 108 top candidate contigs. Contigs are ordered the same as in Figure 2G in the main paper.

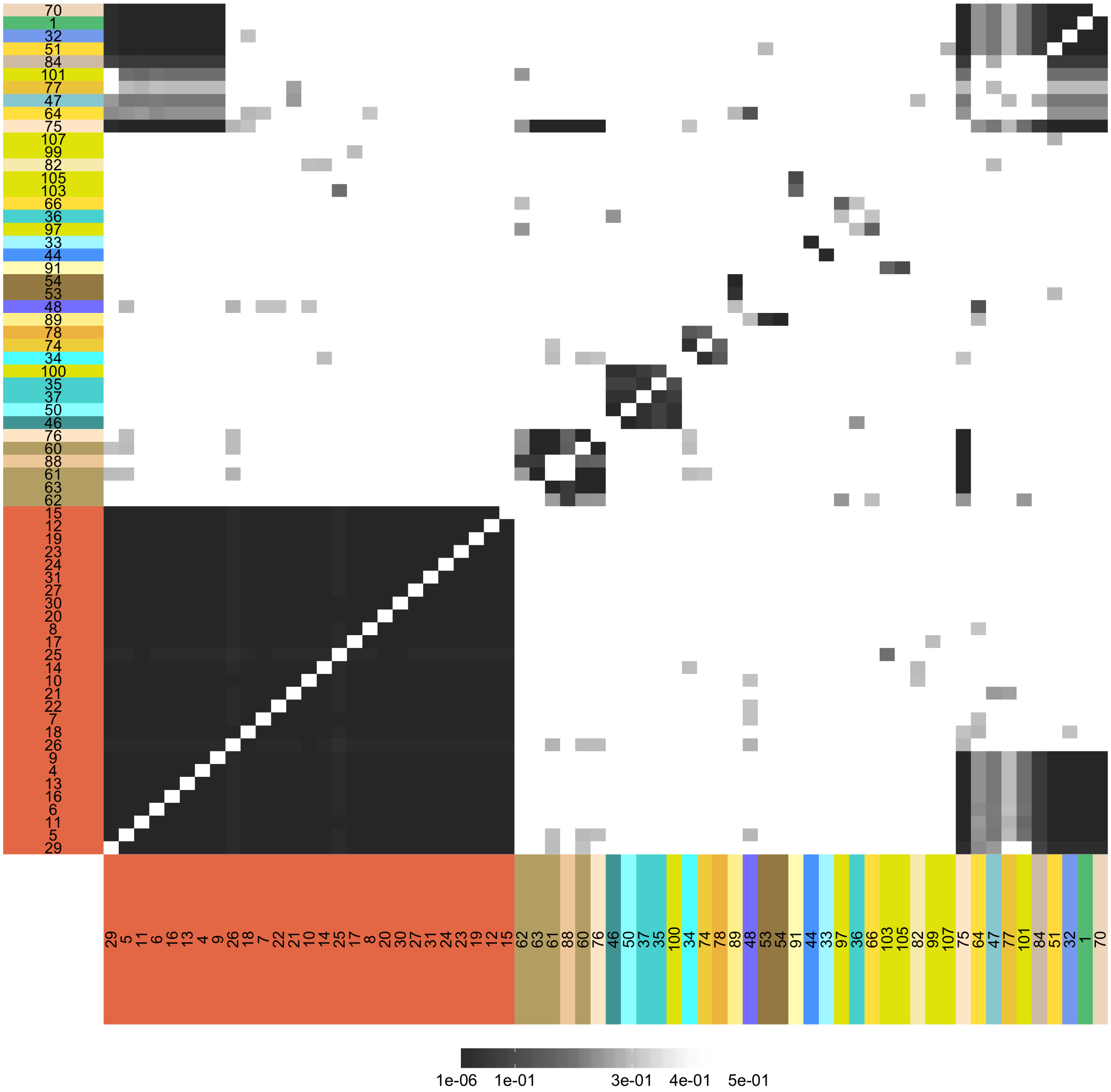

Figure S 8. Recombination heatmap, clustered by recombination rates. The same data as is shown in Figure 3, except re-clustered by recombination rates to more easily see the patterns of physical linkage.

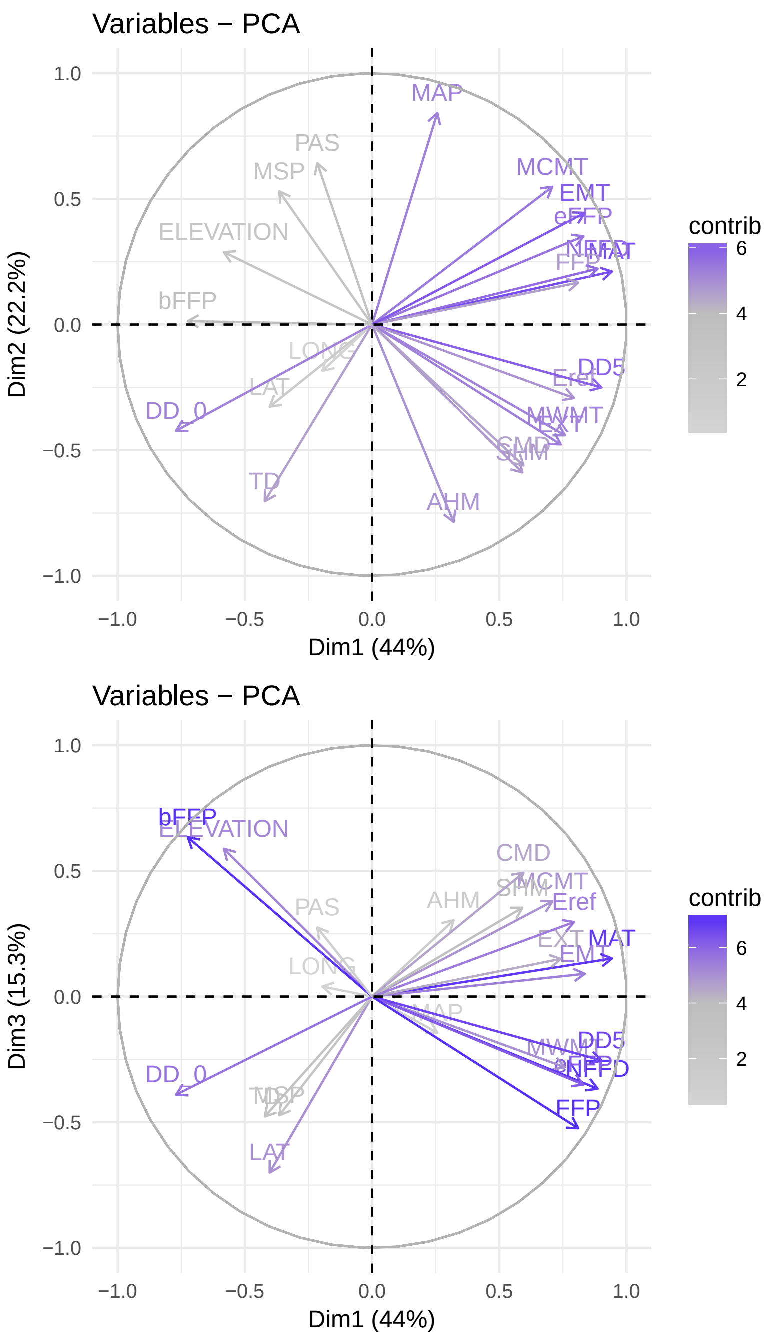

Figure S 9. Loadings of environments onto PC axes. The length and direction of each vector represents the scaled loading of that environmental variable onto the principal components (PC) axis. The color of each vector represents the mean proportion of variance explained by that environment in the two axes plotted.

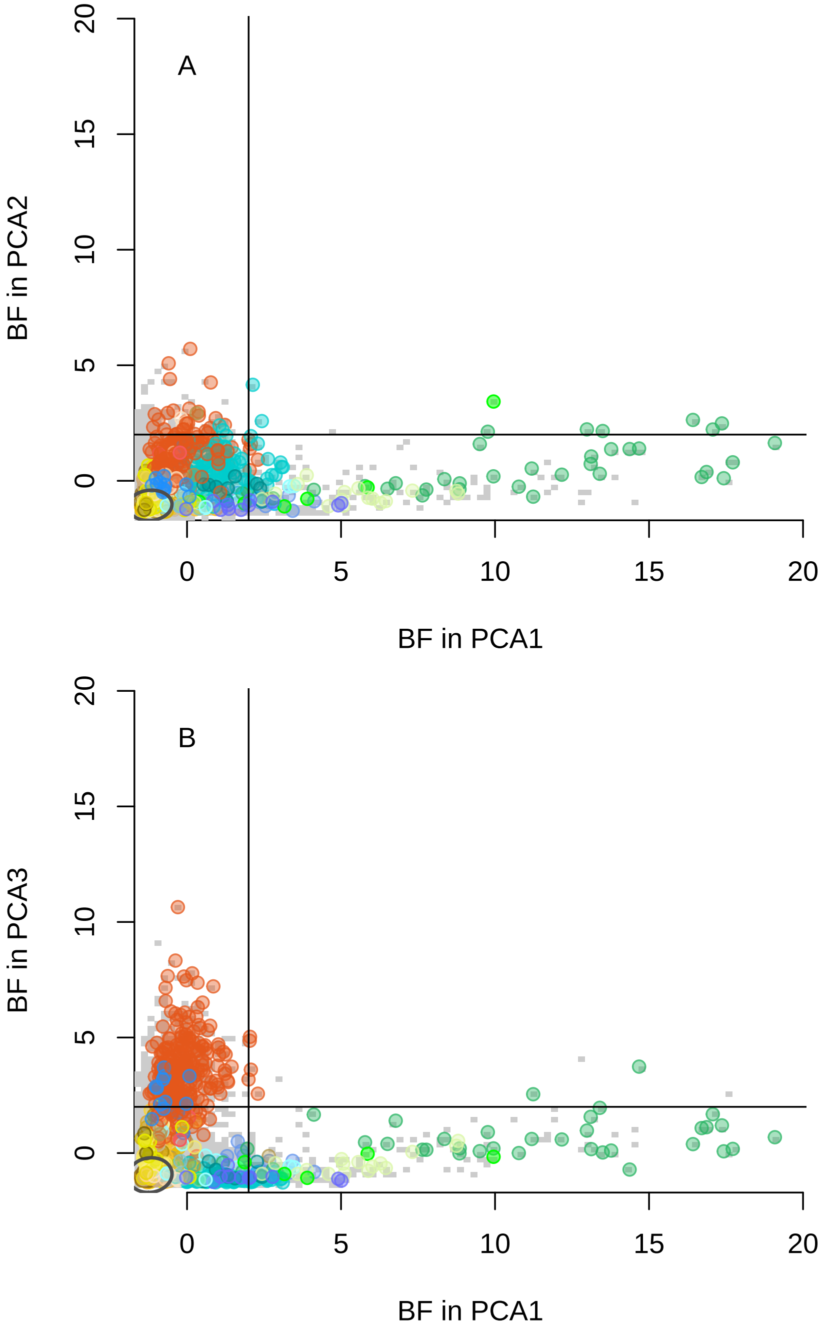

Figure S 10. The distribution of log-10 Bayes Factors for the association between a SNP and a principal components (PC) axis. Each point is a SNP colored according to its co-association module in Figure 2C-F. Vertical and horizontal lines represent criteria for significance, and the black ovals represent the 95% prediction ellipse. Note that candidate SNPs all had Bayes Factor (BF) > 2 with at least one univariate environmental variable.

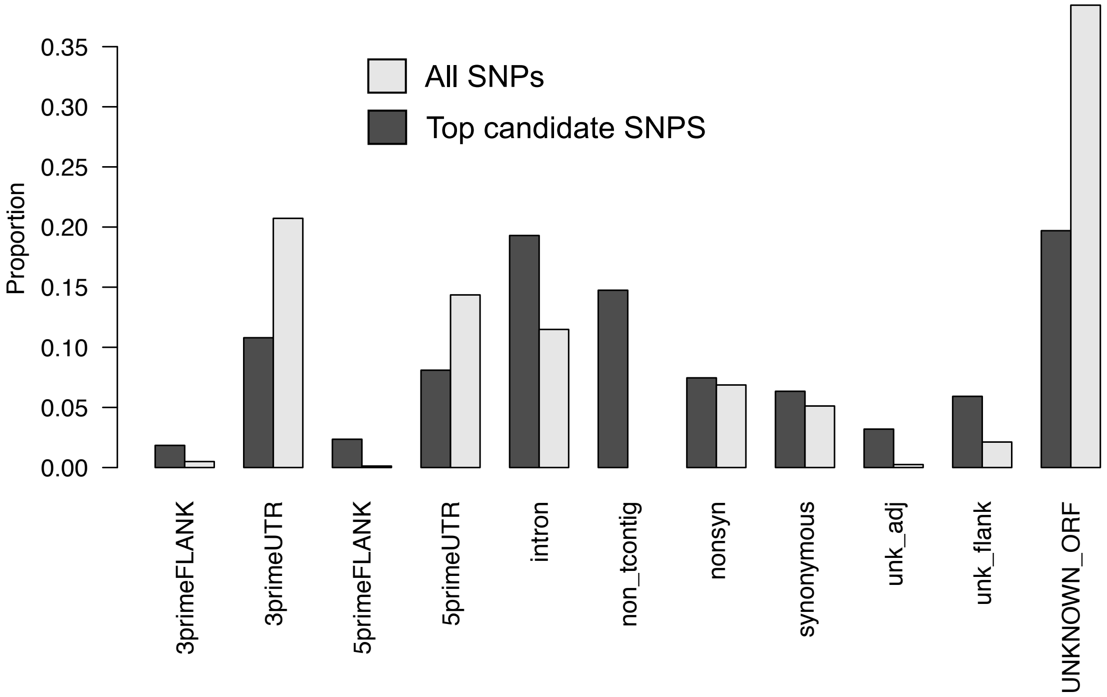

Figure S 11. SNP annotations and genomic features. Proportion of exome SNPs falling into various categories for genomic features compared to in the top candidate list. All loci are shaded in dark grey, while top candidates are shaded in grey. 3primeFLANK: 3' flanking region; 3primeUTR: 3' untranslated region; 5primeFLANK: 5' flanking region; 5primeUTR: 5' untranslated region; non-tcontig: not located in a transcriptomic contig (intergenic); nonsyn: non-synonymous substitution; unk-adj: unknown adjacent region; unk-flank: unknown flanking region; UNKNOWN-ORF: unknown open reading frame.

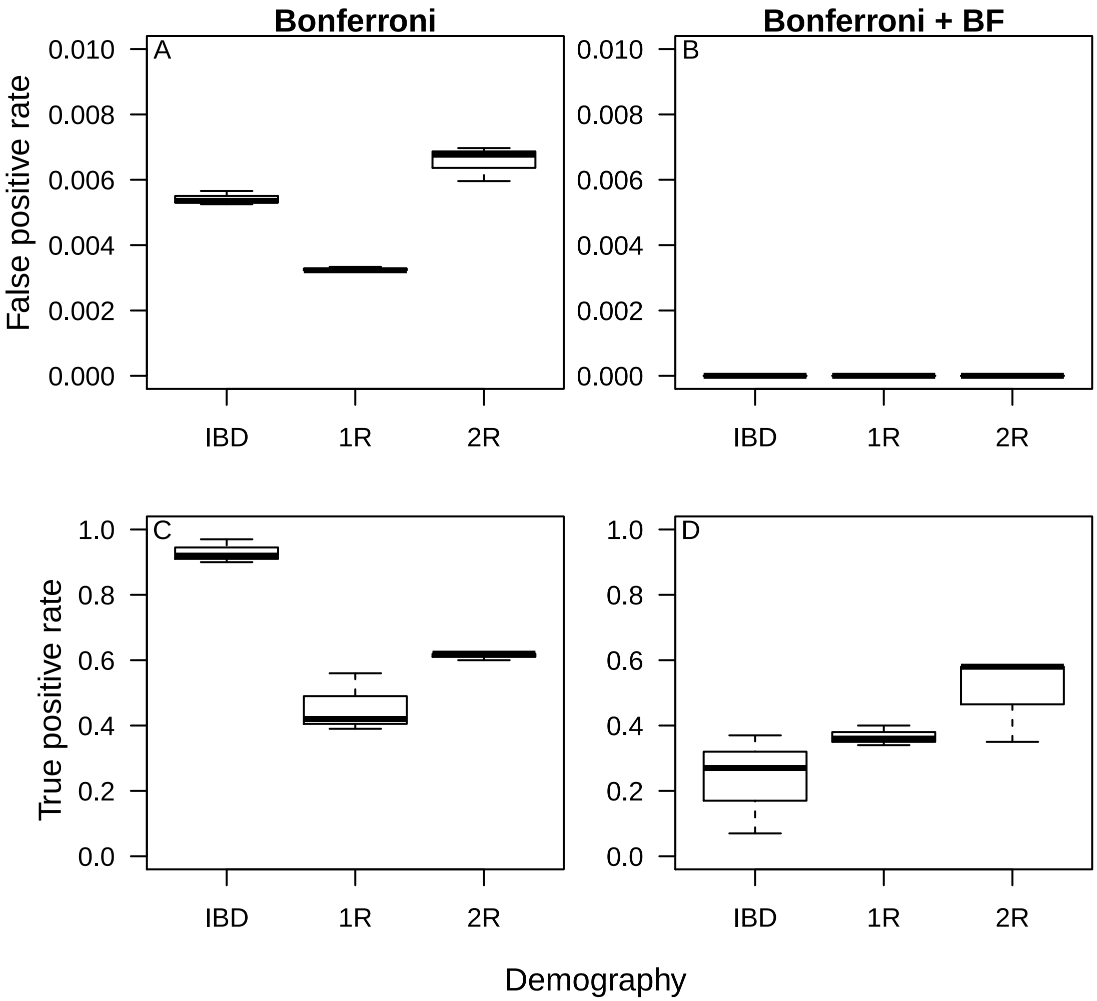

Figure S 12. Box plots of error rates from the simulations given a less stringent criterion (Bonferroni, left) and a more stringent criterion (Bonferroni and Bayes Factors from bayenv2, right). The less stringent criterion was used for the simulations because it had some false positives (A), while the more stringent criterion was used for the empirical data because it did not have any false positives (B). The three demographies are isolation by distance (IBD), range expansion from one refugium (1R), and range expansion from two refugia (2R). While using the more stringent criteria resulted in no false positives, it also reduced the number of true positives (compare C and D), with the most severe reduction under IBD.

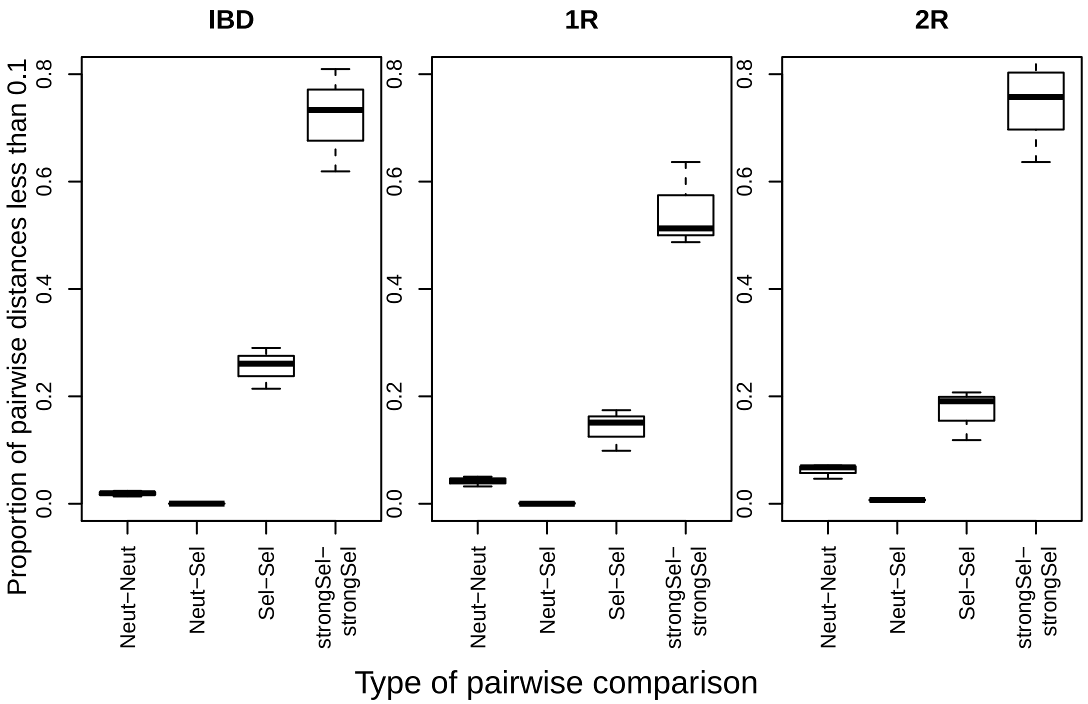

Figure S 13. Boxplots of pairwise distances among loci as a function of selection for simulated data, which was used to evaluate 0.1 as a distance threshold for creating a co-association module. The three demographies are isolation by distance (IBD), range expansion from one refuge (1R), and range expansion from two refugia (2R). For the simulated data, top candidates were chosen as described in the methods. Multivariate Euclidean distance was calculated among the loci based on their associations with environments, and the proportion of pairwise distances above the distance threshold of 0.1 (used for the empirical data) was calculated for each type of comparison. We evaluated four types of pairwise comparisons: neutral loci with each other ("Neut-Neut"), neutral loci with selected loci ("Neut-Sel"), all selected loci with each other ("Sel-Sel"), and only loci under strong selection with each other ($s > 0.1$, "strongSel-strongSel"). A higher proportion of pairwise distances above the threshold indicates that these loci would be more connected to each other in the co-association network.

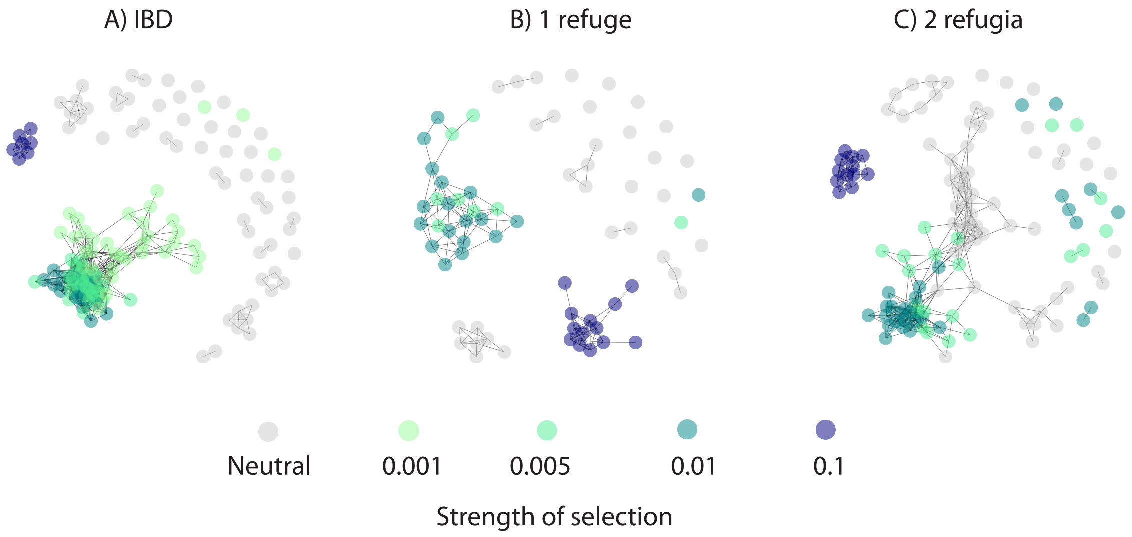

Figure S 14. The simulated datasets were nested within randomly generated selective environments, such that different demographic histories were simulated on the same environmental landscape. For this randomly generated environment, loci simulated under stronger selection had a propensity to cluster differently than loci simulated under weaker selection. To be clear, they still show the same patterns of associations, but the absolute value of the associations was just larger for the loci under strong selection and this caused the creation of a second cluster.
